# Hierarchical multi-task deep learning-assisted construction of human gut microbiota reactive oxygen species-scavenging enzymes database

**DOI:** 10.1128/msphere.00346-24

**Published:** 2024-07-12

**Authors:** Yueyang Yan, Zhanpeng Shi, Yongrui Zhang

**Affiliations:** 1 College of Veterinary Medicine, Jilin University, Changchun, China; 2 Department of Urology, The First Hospital of Jilin University, Changchun, Jilin, China; Third Institute of Oceanography Ministry of Natural Resources, Xiamen, China

**Keywords:** reactive oxygen species-scavenging enzymes, gut microbiota, deep learning, database

## Abstract

In the process of oxygen reduction, reactive oxygen species (ROS) are generated as intermediates, including superoxide anion (O^2−^), hydrogen peroxide (H_2_O_2_), and hydroxyl radicals (OH^−^). ROS can be destructive, and an imbalance between oxidants and antioxidants in the body can lead to pathological inflammation. Inappropriate ROS production can cause oxidative damage, disrupting the balance in the body and potentially leading to DNA damage in intestinal epithelial cells and beneficial bacteria. Microorganisms have evolved various enzymes to mitigate the harmful effects of ROS. Accurately predicting the types of ROS-scavenging enzymes (ROSes) is crucial for understanding the oxidative stress mechanisms and formulating strategies to combat diseases related to the “gut–organ axis.” Currently, there are no available ROSes databases (DBs). In this study, we propose a systematic workflow comprising three modules and employ a hierarchical multi-task deep learning approach to collect, expand, and explore ROSes-related entries. Based on this, we have developed the human gut microbiota ROSes DB (http://39.101.72.186/), which includes 7,689 entries. This DB provides user-friendly browsing and search features to support various applications. With the assistance of ROSes DB, various communication-based microbial interactions can be explored, further enabling the construction and analysis of the evolutionary and complex networks of ROSes DB in human gut microbiota species.

**IMPORTANCE:**

Reactive oxygen species (ROS) is generated during the process of oxygen reduction, including superoxide anion, hydrogen peroxide, and hydroxyl radicals. ROS can potentially cause damage to cells and DNA, leading to pathological inflammation within the body. Microorganisms have evolved various enzymes to mitigate the harmful effects of ROS, thereby maintaining a balance of microorganisms within the host. The study highlights the current absence of a ROSes DB, emphasizing the crucial importance of accurately predicting the types of ROSes for understanding oxidative stress mechanisms and developing strategies for diseases related to the “gut–organ axis.” This research proposes a systematic workflow and employs a multi-task deep learning approach to establish the human gut microbiota ROSes DB. This DB comprises 7,689 entries and serves as a valuable tool for researchers to delve into the role of ROSes in the human gut microbiota.

## INTRODUCTION

In the intricate interplay between gut microbiota and human health, reactive oxygen species (ROS)-scavenging enzymes (ROSes) generated by gut microbiota play a pivotal role. ROS constitute a group of highly reactive oxidative molecules, including superoxide radicals, peroxyl radicals, hydroxyl radicals, and hydrogen peroxide radicals (1). These ROS can originate from exogenous sources, termed the exposure group, such as ultraviolet radiation, chemotherapy drugs, environmental toxins, ionizing radiation, and inflammatory cytokines (2). Endogenous sources encompass mitochondria, lipoxygenases, nitrogen oxides, and peroxisomes. When ROS production exceeds normal levels or clearance mechanisms falter, oxidative stress ensues. Uncontrolled generation of ROS is associated with several health issues, including inflammatory bowel disease, ileitis, gut infections, ischemic intestinal injury, and colorectal cancer (3). Additionally, elevated ROS levels are linked to aging and age-related diseases (4). To prevent or mitigate ROS-induced oxidative stress, gut microbiota deploy various ROS defense mechanisms. Currently, the known ROSes are primarily categorized into nine major classes, encompassing 26 different types of enzymes (5). These classifications reflect their critical roles in countering oxidative damage within cells, protecting tissues, and regulating biochemical reactions.

Databases (DBs) like COG (6), KEGG (7), eggNOG (8), and SEED (9) are invaluable for decoding gene functionality, each bringing its unique advantages. For instance, the COG DB, which is grounded in complete microbial genomes, excels at identifying orthologs and paralogs due to its reliance on 4,631 orthologous groups. On the other hand, KEGG stands out for its ability to link genes to specific pathways. SEED adopts a subsystem strategy to enhance the accuracy of orthologous gene annotations, moving away from conventional methodologies. EggNOG provides a comprehensive view by offering annotations and predicted functional categories for genes based on orthology relationships. While ROSes have not been systematically compiled in dedicated DBs, they have been individually gathered, potentially limiting insights into the antioxidative capabilities of microbial communities in the human gut microbiota. Additionally, existing research has often relied on a restricted selection of reported ROSes; the comprehensive mining and integration of novel ROSes-related genes to establish a more complete DB remain areas for further exploration.

While these DB are extensively utilized, their dependence on sequence similarity for annotations can lead to inaccuracies (10). This is because some protein sequences might be misannotated due to their low sequence similarity or the application of high threshold values in the annotation process. To overcome these limitations, adopting a more integrated approach becomes crucial. Leveraging deep learning for function prediction presents a significant advantage by its capacity to unearth deeper patterns and features within protein sequences (11).

In this study, our aim is to address the aforementioned issues through a comprehensive approach. Firstly, we have devised a systematic workflow comprising gene collection, mining, and evolutionary analysis modules to construct the ROSes DB for the human gut microbiota. In the gene collection module, apart from the intestinal ROSes genes found in common public DBs, we meticulously curated genes across diverse bacterial domains of the gut microbiota. Gene prediction and basic annotation were performed to identify ROSes candidates. The mining module integrates our previous ROSes-FINDER algorithm (12), employing machine learning techniques (xgboost), convolutional neural networks (CNNs), and deep neural networks, along with a voting-based approach. This combination, incorporating protein annotation, functional analysis, and sequence characteristics, allows for the reannotation and exploration of ROSes entries. Subsequently, in the evolutionary analysis module, a diverse analysis of human gut microbiota ROSes genes was conducted. These efforts culminated in the creation of the human gut microbiota ROSes DB (http://39.101.72.186/), which offers user-friendly browsing and search functionalities to support various applications.

## RESULT

### DB data collection and architecture design

**Fig 1:**
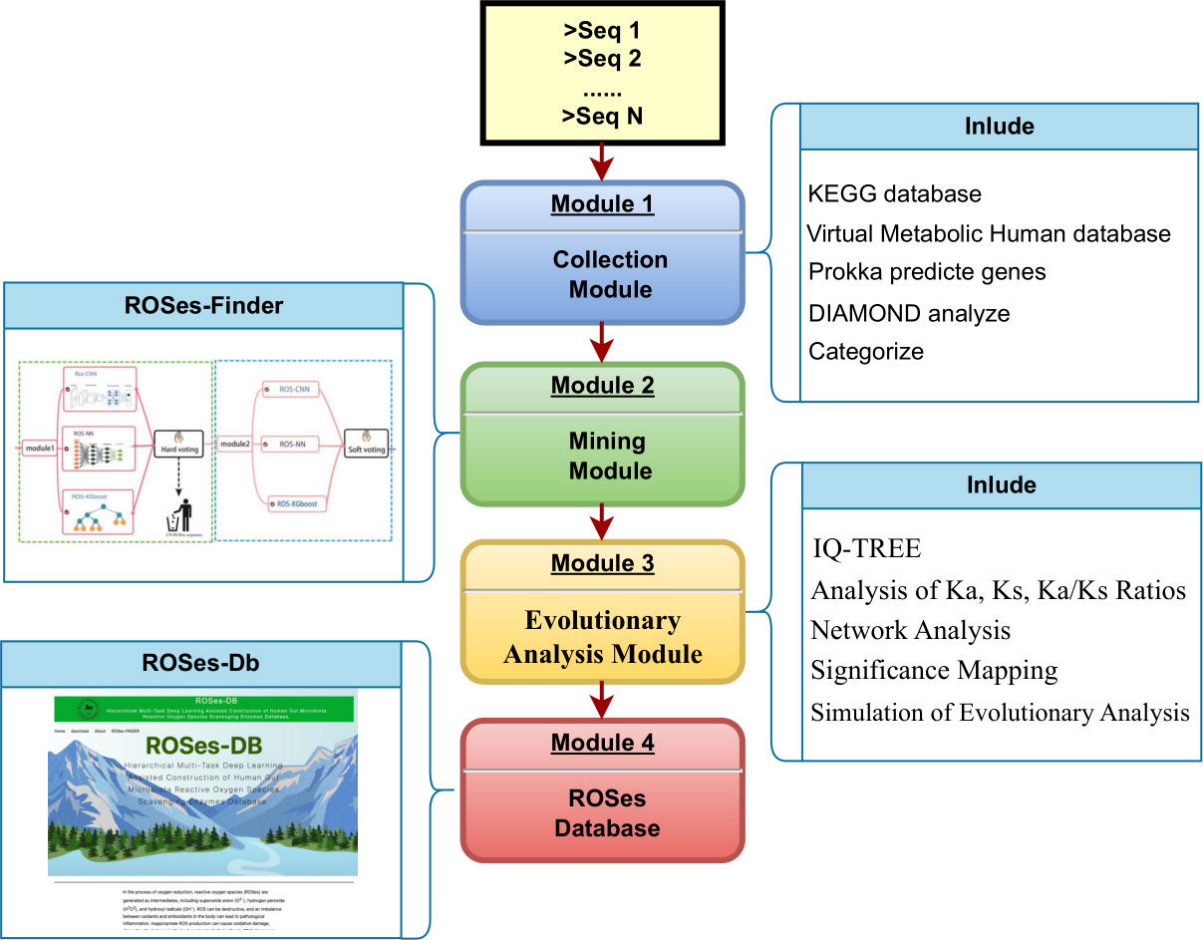
ROSes DB construction workflow. (A) Sequence retrieval and screening: initially, protein sequences are retrieved using the BLASTP algorithm to identify potential target proteins. (B) ROSes identification and classification: using a composition of deep learning models, proteins identified in the first step are analyzed for ROSes identification. If recognized as ROSes, they undergo secondary prediction. In this stage, a multi-task deep learning model is trained to predict specific ROSes categories. (C) Integration and network establishment: evolutionary information of the identified ROSes, including gene mutation data, is integrated and utilized to establish the ROSes network.

We developed a ROSes mining workflow leveraging three hierarchical multitask classifiers based on machine learning and deep learning algorithms, aimed at constructing a ROSes repository for the human gut microbiota. The workflow is divided into four modules, starting with the collection module where we gathered KEGG oxidative-reduction pathways. Through manual searches for gene names and annotation lists related to antioxidant gene families, we generated a refined set of 59,893 ROSes sequences from the DB as reference gene sequences. In the mining module, 818 gut microbiota species from the Virtual Metabolic Human DB (https://www.vmh.life/) (File S1) were considered as representatives of human gut microbiota. Potential ROSes were identified for each human gut microbiota gene based on its sequence identity (10%) and *E* value (1^e−3^) with the reference sequences (File S2), aiming for comprehensive coverage of reported ROSes entries. These preliminarily screened sequences underwent further evaluation using the ROSes-FINDER (accuracy is 94.29%, and precision is 99.01%), where three different algorithms (Xgboost, CNN, and NN) were employed to construct classifiers, modeling and analyzing potential ROSes using a hard voting algorithm (Fig. 1).

A refined set of 7,689 ROSes sequences (File S3) was generated from the initial DB, following the principle that a gene is considered ROSes only if two or more algorithms corroborated its classification as ROSes. These genes were then categorized into “high” (predicted as true by three algorithms) and “medium” (predicted as true by two algorithms) annotation confidence categories. The identified ROSes underwent further classification and annotation based on the soft voting algorithm, culminating in the ROSes DB. This database comprises 26 ROSes categories, with over 19% of genes belonging to thioredoxin_1 (1,503), followed by thioredoxin reductase (1,466), 7.2% to glutathione peroxidase (558), and 5% to thiol peroxidase. Finally, through the evolutionary analysis module, a comprehensive network and evolutionary analysis of the 7,689 genes were performed.

### The distribution of ROSes-active enzymes in the human gut microbiota

**Fig 2:**
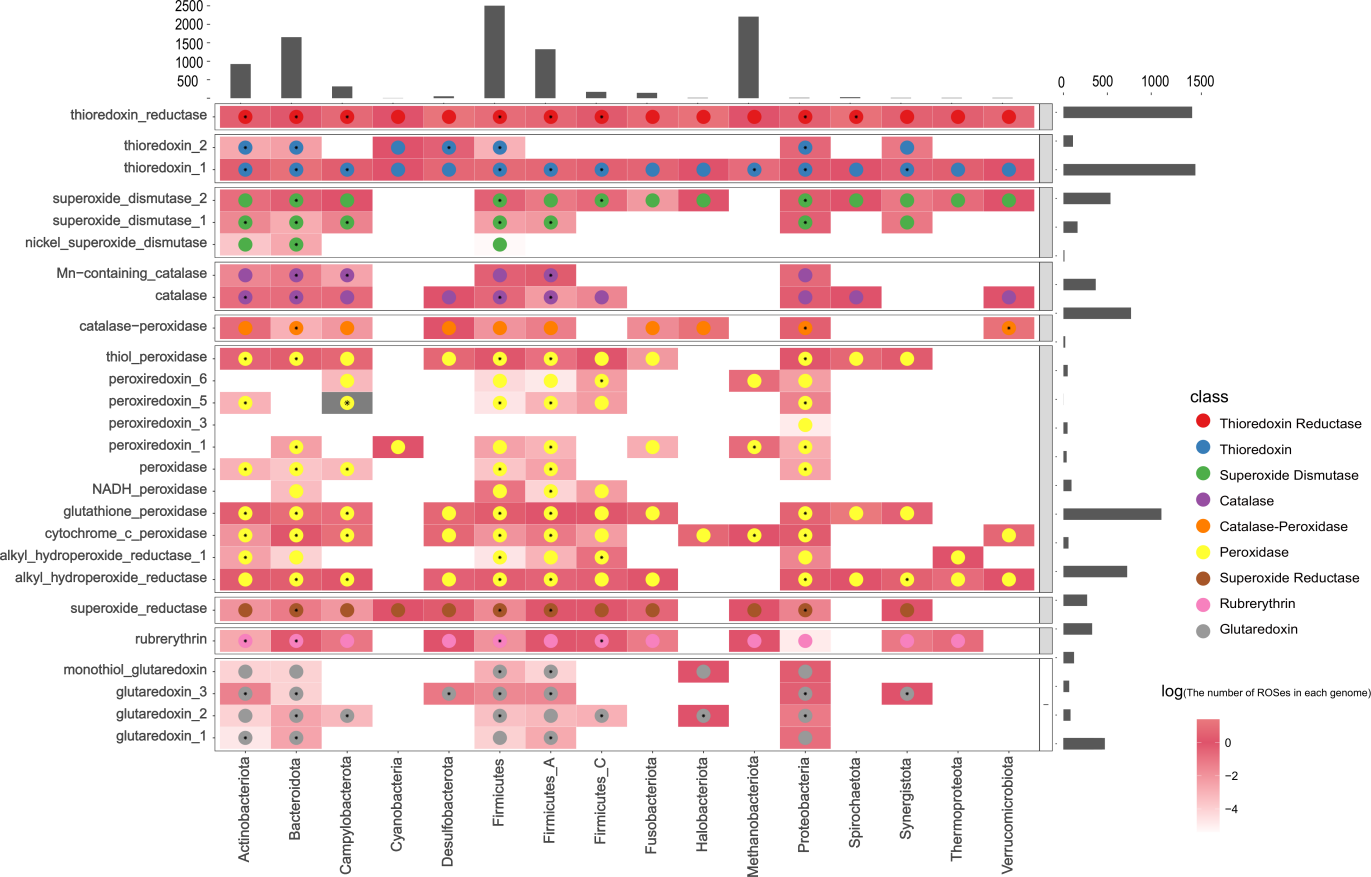
A multi-information heatmap of ROSes detected in the human gut microbiota using ROSes DB. Each column represents a phylum, with the histogram at the top displaying the number of genomes in each phylum. Rows represent ROSes protein families grouped by their functional types (labels on the left).The grayscale background of each square represents the number of homologs detected in the reference protein families. An asterisk indicates the best homolog with a BLAST *E* value < 1e−10.

We summarized the distribution of 26 known ROSes defense enzymes and assessed their presence and prevalence within bacterial phyla (Fig. 2). This analysis encompassed a total of 818 bacterial genomes representing 16 different phyla. There was significant variation in the distribution of ROSes defense mechanisms across the bacterial domain. The most common enzymes, thioredoxin_1 and thioredoxin reductase, were found in all phyla, followed by glutathione peroxidase and thiol peroxidase. These enzymes play roles in intracellular thiol/disulfide balance and catalytic reduction of oxidized proteins through disulfide bonds or sulfenic acids.

In the Bacteroidota phylum, enzymes such as NADH peroxidase and thioredoxin reductase show a higher occurrence, with an average of 0.71 of these specific genes present per genome. This implies that the Bacteroidota phylum may have a greater capacity for adapting to oxidative stress. Conversely, the Firmicutes phylum exhibits a broader array of ROSes, including Mn-containing catalase, NADH peroxidase, and thioredoxin reductase, with a higher gene density averaging 1.22 per genome. Firmicutes might employ more diverse strategies and adaptations in response to oxidative stress. Meanwhile, the Synergistota phylum shows a comparatively lower gene density for certain ROSes, such as alkyl hydroperoxide reductase and thioredoxin reductase, averaging at about 0.56 genes per organism. This lower gene density may indicate a diminished capacity of the Synergistota phylum to adapt to oxidative stress.

At the species level, each genome contains an average of nine ROSes, with significant variation in the number of ROSes across different microbes. Over 20% of the microorganisms did not have annotated ROSes. The genome of Actinomyces georgiae contains 31 ROSes. Moreover, actinobacteria are considered to be the most potent sources of antioxidants, antibiotics, and other bioactive compounds (13). We have provided a table of the ROSes present in all species in File S4.

### Revealing oxidative stress pathways in the human gut microbiota: construction of a ROS protein interaction network

**Fig 3:**
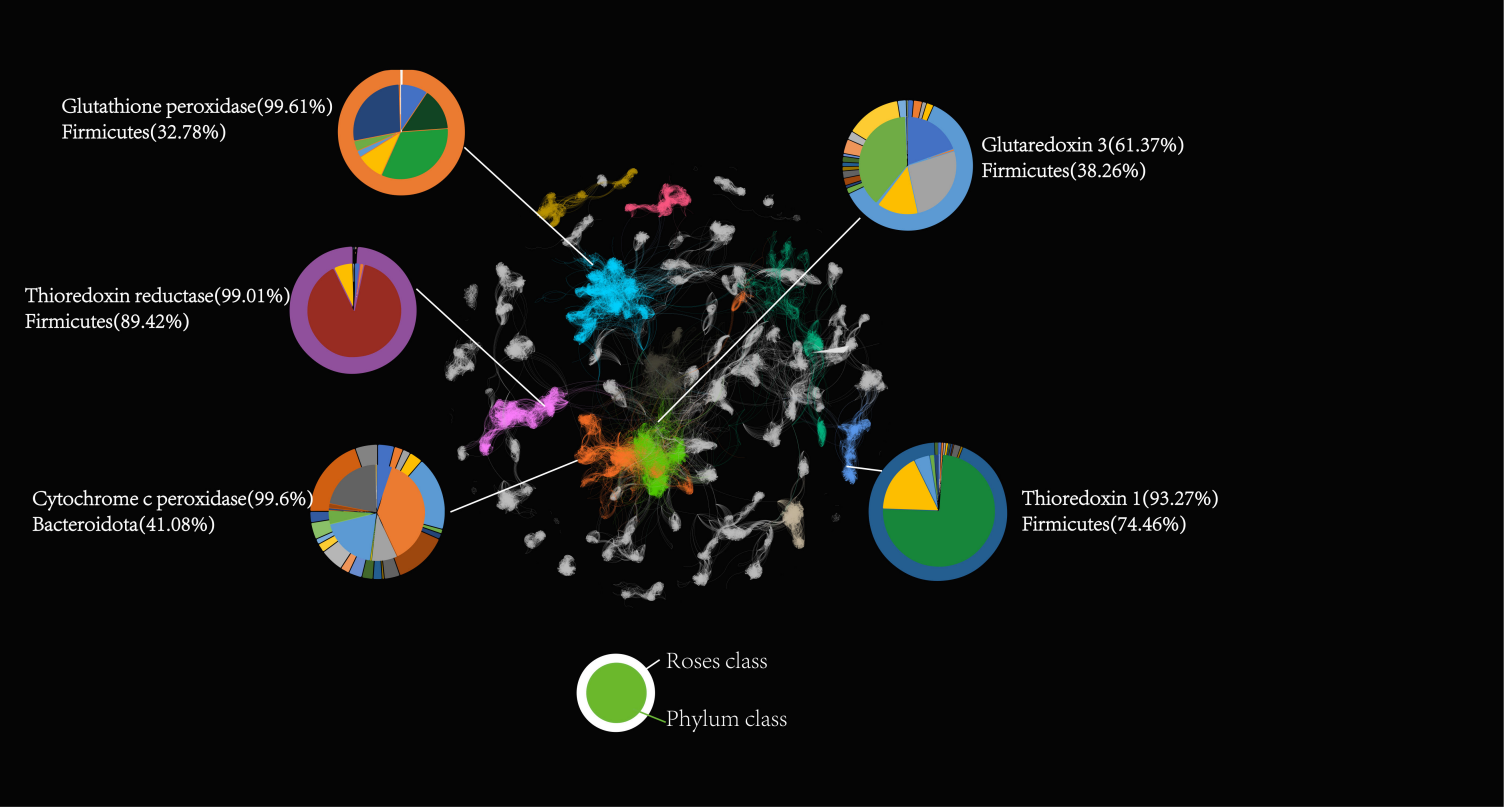
ROSes Network. The outer circle represents each ROSes class, while the inner circle represents the corresponding bacterial phylum. Nodes of different colors represent different ROSes classes, and the lines connecting the nodes indicate sequence similarity. Cluster analysis was conducted on these nodes employing a method predicated on sequence similarity, wherein distinct clusters are delineated by varying colors.

We assessed the pairwise similarities among ROSes proteins and, from these similarity matrices, crafted a ROSes network (Fig. 3). In this network, nodes symbolize protein sequences, and edges reflect the similarities between these sequences. We also developed network models on random combinations (Fig. S1). A comparative analysis of these networks revealed a pronounced preference for functional gene clustering over species-based clustering within the sequence similarity network. This indicates that, within this network, the functional attributes of genes and their involvement in metabolic pathways hold greater significance than the classification based on species. Among the top four principal clusters of the sequence similarity network, we pinpointed the most prominent clusters to be glutathione peroxidase, with subsequent clusters comprising thioredoxin reductase and thioredoxin. These clusters encompass a set of genes with substantial functional interconnectedness, crucial for sustaining the intracellular redox equilibrium and shielding cells from oxidative harm. Specifically, glutathione peroxidase, a pivotal ROSes, counters the cellular damage inflicted by ROS, whereas thioredoxin and thioredoxin reductase are integral to the core of cellular redox reactions. Conversely, the network formed from random combinations displayed a dispersion of species and gene functions across clusters, lacking a clear pattern of specific clustering.

**Fig 4:**
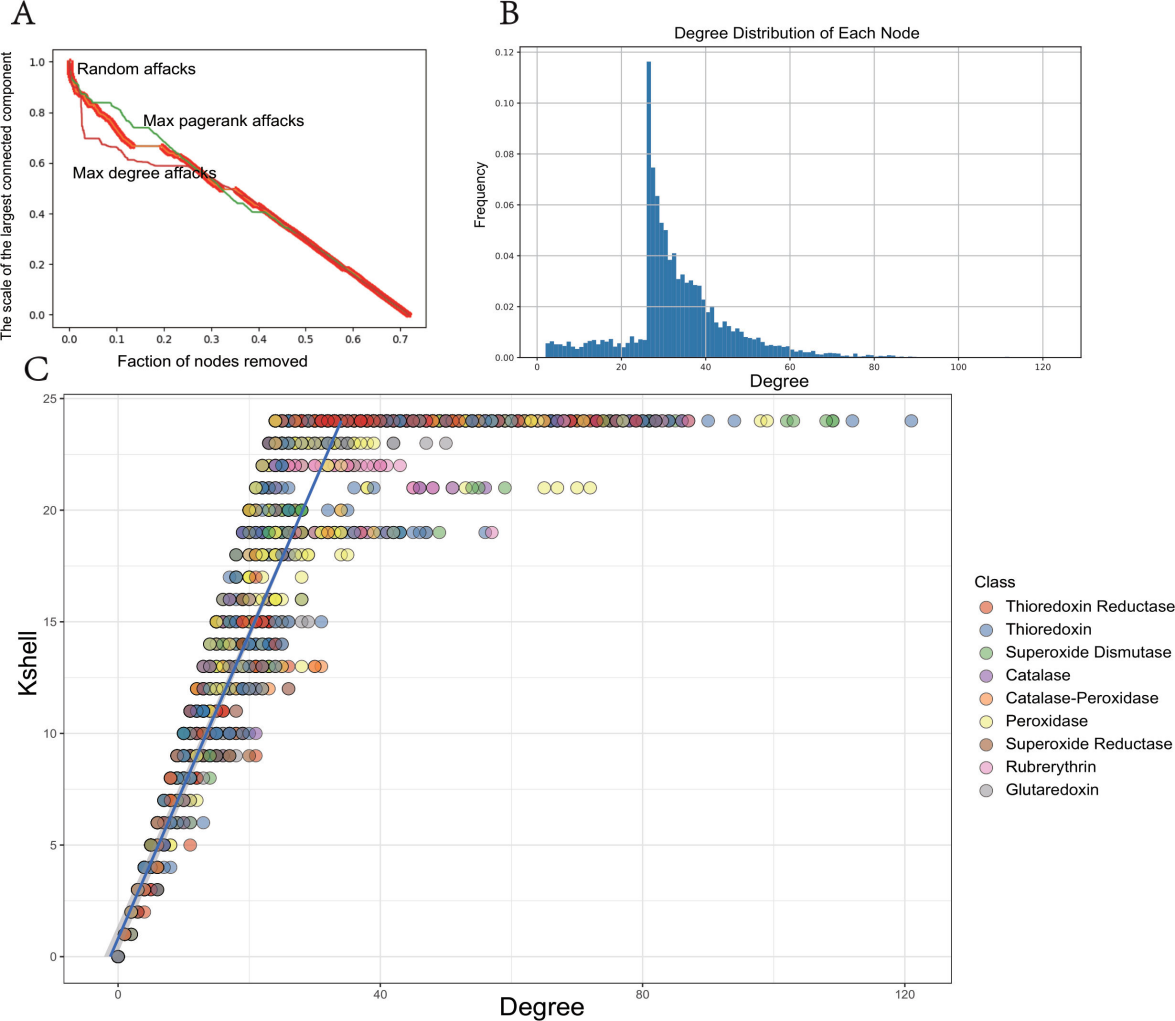
Network topology and robustness. (A) Relationship between the proportion of removed nodes and the size of the largest connected component; (B) degree distribution of each node; (C) schematic representation of network stratification using K-shell decomposition method.

Figure 4 clearly demonstrates that targeted attacks are more effective than random attacks in simulation, highlighting the ROSes network’s strong resistance to random attacks. In simulations with targeted attacks, the network’s stability rapidly declines, showing a significant downward trend, emphasizing the superiority of importance ranking techniques based on the maximum degree attacks and maximum PageRank attacks (Fig. 4A). The node degree exhibits a sharp decay distribution, skewed toward nodes with low connectivity(Fig. 4B). This indicates the presence of a few highly connected core nodes in the ROSes network, while the majority of nodes have low connectivity. This characteristic may reflect the complex interaction patterns among protein sequences in the ROSes network, where only a few nodes exhibit high correlations, while most nodes are relatively less interconnected.

K-shell decomposition is a network analysis method that categorizes nodes into layers based on their connectivity, revealing the core periphery structure of complex networks. By iteratively removing nodes with the least connections, it identifies the most interconnected nodes as the network’s core (14). The K-shell technique was applied to examine the hierarchical structure of the ROSes network (Fig. 4C). This process was repeated until the most centralized core nodes were identified. In contrast, nodes in the network’s periphery are more dispersed, with their averages significantly lower than those close to the core nodes. Statistical data indicate that inner layer nodes might serve as more effective information transmitters. Additionally, we discovered that the ROSes network’s topology resembles that of the internet network. While only 0.5% of nodes constitute the core in internet networks, in the ROSes network, a mere 0.1% of nodes form the core. This finding provides new insights into the internal structure and information transmission methods of complex microbial systems.

### Capturing evolutionary information of ROSes

**Fig 5:**
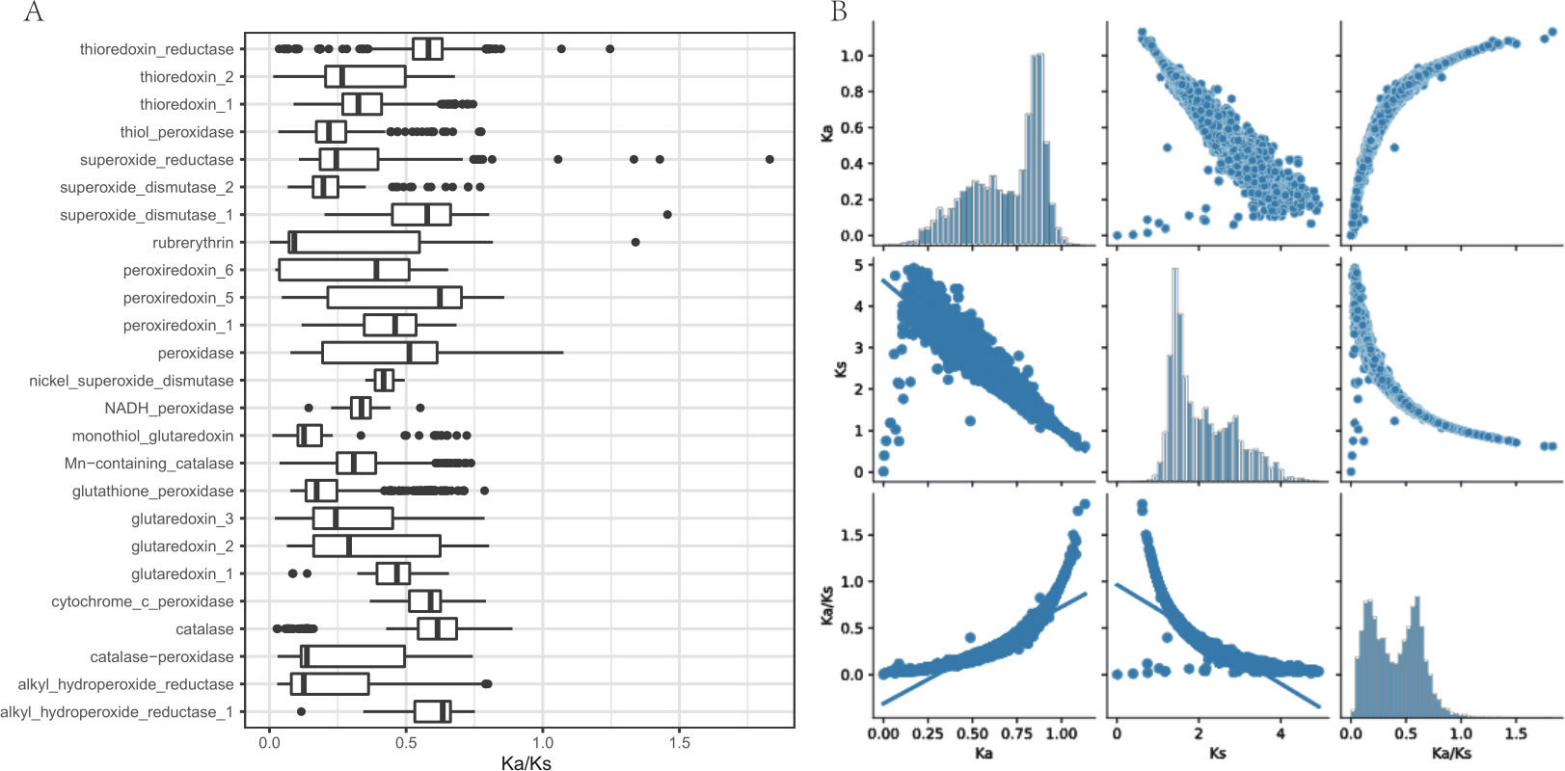
Evolution of ROSes. (A) Boxplots depicting the distribution of Ka (non-synonymous mutations)/Ks (synonymous mutations) ratios for different ROSes genes. (B) Distribution and correlation (Spearman rank correlation coefficients) of Ka, Ks, and Ka/Ks ratios in ROSes.

The analysis of the evolutionary rates of ROSes genes reveals their relatively conservative nature during the evolutionary process (Fig. 5A). The average Ka rate is 0.688, indicating a relatively slow occurrence of amino acid replacements, while the average Ks is 2.11, suggesting frequent synonymous mutations. The calculated average Ka/Ks ratio is 0.401, indicating strong negative selection pressure on ROSes genes, reflecting their influence under conservative selection during evolution (Fig. 5B).

Further analysis of selection pressure reveals specific evolutionary forces acting on ROSes genes. Specifically, the correlation coefficient between Ka/Ks is 0.95, indicating a strong positive correlation between non-synonymous mutations (Ka) and amino acid replacement rate (KA). This suggests that these genes might have undergone similar selection pressures during evolution, leading to similar changes at the amino acid level. However, the correlation coefficient between Ka/Ks and KS is 0.37, indicating a weaker positive correlation between Ka and Ks. This implies that these genes have experienced higher rates of change during evolution but have maintained relative stability in Ks.

At the level of the ROSes gene family, catalase, alkyl hydroperoxide reductase 1, and thioredoxin reductase exhibit higher average Ka/Ks ratios, indicating strong selection pressure on these genes during evolution. In contrast, alkyl hydroperoxide reductase, superoxide dismutase 2, and monothiol glutaredoxin have lower Ka/Ks ratios, suggesting weaker selection pressure. This likely reflects their crucial roles in maintaining cellular stability and resisting oxidative stress.

**Fig 6:**
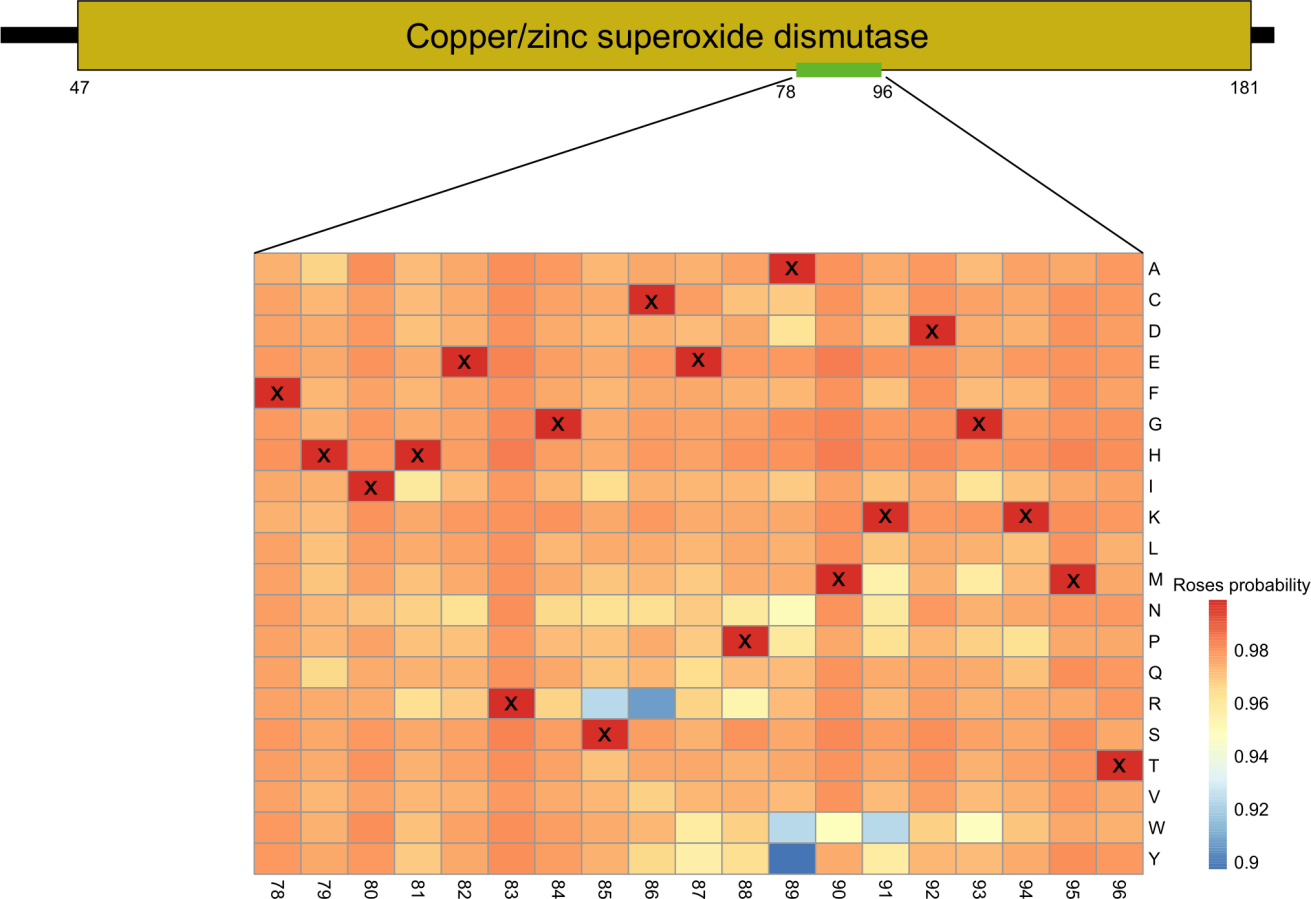
Evolutionary analysis of the superoxide dismutase sequence. The conserved domain of the superoxide dismutase sequence (positions 47–181) is displayed, with the most crucial signal extracted from positions 78–96 for evolutionary analysis. Each column represents a position in the sequence, while rows indicate amino acid mutations. Mutated sequences were input into the model to determine the probability of each sequence being a ROSes within the sequence, with corresponding probability values filled into the significant plot at their respective positions.

To further investigate the evolutionary directions of ROSes, we conducted significance mapping (mutation mapping) analysis (Fig. 6). For each amino acid in ROSes sequences, we mutated it to different amino acids and fed the mutated sequences into the trained model to obtain the prediction probability of the mutated sequence being a ROSes. Since each amino acid can mutate into 20 different amino acids (including itself), the significance map for each ROSes will have dimensions of L × 20, where L is the sequence length. Through this mutational analysis, we were able to identify the conserved sites predicted by our model. Taking superoxide dismutase sequence as an example, we constructed a significance map aligned with it. We selected the most significant signal in the mutational map, namely, 68 (A to Y), and applied this mutation to the protein sequence. The comparative results are shown in Fig. 6. Despite mutating only one amino acid, significant changes occurred in the local structure and environment, indicating the importance of this conserved site. To capture evolutionary information of specific sequences, high values in the matrix represent highly conserved positions. This method provides an in-depth understanding of the conservation and evolutionary characteristics of ROSes sequences, offering new insights into the development of antioxidant enzymes and the exploration of antioxidant stress mechanisms.

## DISCUSSION

ROS (free radical forms O^2•−^, superoxide radical; OH^•^, hydroxyl radical; ROO^•^, peroxyl; and RO^•^, alkoxyl, and non-radical forms O^2^, singlet oxygen, and H^2^O (2), hydrogen peroxide) are inevitable companions of aerobic life, playing a crucial role in gut health. However, the excessive production of ROS can cause severe damage to biomolecules (15).

We deployed a hierarchical multitask deep learning framework to collect, formulate, train, and evaluate an innovative computational resource aimed at identifying and annotating ROSes from metagenomic data. This deep learning approach has outperformed traditional best-hit methods in terms of accuracy, effectively addressing the limitations imposed by stringent cutoff values and significantly lowering the incidence of false negatives. Our prior research (12) reveals that the ROSes-FINDER achieves a false-positive rate of 1.17%, a false-negative rate of 5.68%, and an overall accuracy rate of 94.29%. The success of ROSes-FINDER is attributed to its deep learning-driven predictive capability. The deep learning approach demonstrated superior accuracy compared to the commonly used best-hit approach and overcame limitations associated with strict cutoffs, substantially reducing false negatives. The versatility of ROSes-FINDER allows for its application not only in analyzing gut microbiota but also in broader environmental settings, including water sources and soil metagenomes, by enabling precise identification of ROS in diverse microbiomes.

Various interactions based on ROSes play a significant role in the regulation of homeostasis, metabolism, and immune responses in the human intestinal system (16). Therefore, constructing a comprehensive ROSes DB for the human gut microbiota is crucial for enhancing the predictability of gut microbiology and developing potential therapies for various intestinal diseases. In this work, we developed a systematic workflow, including collection, expansion, and mining modules, to construct a comprehensive ROSes repository for the human gut microbiota. Algorithms such as Xgboost, RNN, and NN, along with protein annotation and functional analysis, were combined to enhance the efficiency of data collection and mining. As a result, we established ROSes DB (http://39.101.72.186/), featuring browsing and search functionalities, containing 7,689 ROSes entries from 818 human gut microbiota species.

With the assistance of ROSes DB, users can explore numerous ROS-based interactions within various microbial communities, based on different ROS defense characteristics. We have constructed a ROSes-net to visualize and decipher the complex interactions within the human gut microbiota based on ROSes. Similar to the functional hierarchy observed in genetic interaction networks from yeast to humans, the clusters in our ROSes network form a hierarchical structure consistent with the functional hierarchy observed in genetic interaction networks in yeast cells; genes within the same cluster are enriched with closely related functions. We observed that clustering in ROSes network often occurs at the type level rather than the species level. Furthermore, the interactions in network between microbes support the concepts of diversity and redundancy in microbial ROSes, contributing to the stability of microbial redox systems (5).

ROSes DB not only identifies ROSes but also recognizes crucial sites. We found that ROSes excel in capturing evolutionarily conserved amino acids. By employing significance plots (17), we identified sites with high predicted probabilities (indicating strong conservation) under different amino acid mutations. These sites likely play vital roles in evolution and exhibit conservation in both structure and function of proteins. ROSes play a pivotal antioxidative role in organisms. If the amino acid sequences of these enzymes are crucial for their functionality, natural selection tends to preserve these sequences to maintain enzyme activity. During evolution, the functionality of ROSes may not have significantly changed. This conservation suggests that the fundamental functions of ROSes are similar among different species or even among different individuals within the same species.

Additionally, our ROSes DB inevitably contains some false-positive relationships. On the other hand, the ROSes predicted based on the DB will be deemed as “possibilities” rather than certainties and still require experimental validation. The purpose of constructing this data set is to collect as many potential entries related to ROSes as possible, let alone confirming their direct relevance to ROSes. In this work, to explore more potential ROSes entries, we combined manual screening, BLASTP-based extension (18), and machine learning/deep learning-based classification to minimize false positives as much as possible. We present the first ROSes DB, allowing users to initially consider the “possibilities” for various applications, enabling them to focus their efforts on experimental validation afterward.

### Conclusion

The large-scale data established in this study offer invaluable insights into potential functional details based on ROSes interactions among different intestinal microbiota, accessible through our ROSes DB. At the strain level, ROSes DB provides user-friendly data searches, aiding the scientific community in exploring and operating the ROSes DB system. This understanding can unveil how cells protect themselves from oxidative stress injuries, contributing not only to recognizing the body’s self-repair mechanisms but also offering crucial clues for disease prevention, drug development, and advancements in medical treatments.

## MATERIALS AND METHODS

This study aims to establish a meticulously curated small-scale DB for the rapid and accurate analysis of ROSes genes from shotgun metagenomic data of the human gut microbiota. The envisioned DB is expected to possess the following features: (i) it should represent the most recent knowledge about ROSes genes, (ii) all gene families included in the DB should be presented with clarity and precision, and (iii) efforts should be made to minimize false-positive assignments in DB searches. To achieve these objectives, a three-step module was developed.

### Collection module

KEGG, one of the most comprehensive DB for ROSes analysis in metagenomics, was utilized. It is renowned for its high quality and maintenance. We referenced the KEGG oxidative-reduction pathways and manually extracted gene names and annotation lists related to antioxidants (File S5).

In this study, a total of 818 gut microbiota species from the Virtual Metabolic Human DB (https://www.vmh.life/) (19) were considered as representatives of the human gut microbiota. Gene prediction was performed using Prokka (20), and phylogenetic analysis was conducted. DIAMOND (21), a program similar in performance to BLAST but faster, was utilized. Based on DIAMOND results, the predicted genes from the human gut microbiota were aligned to those of the ROSes DB. Genes were categorized based on their sequence identity and potential to exhibit ROSes properties using the following criteria, defined as annotation factors according to methods described by DeepARG (22).

### Mining module

We previously developed ROSes-FINDER to validate and explore potential ROSes. This framework employs a hierarchical prediction strategy, deploying a tiered structure for ROSes scavenging enzyme classification. Given a protein sequence, ROSes-FINDER (12) (https://github.com/alienn233/ROSes-Finder) first classifies it as a ROSes or non-ROSes. If the input sequence is a ROSes, we predict which ROSes category it belongs to. The framework integrates three component methods, using a voting-based approach to simultaneously predict multiple ROSes properties. It can identify whether a given protein sequence is a ROSes and determine its type. The three component methods used in this framework are ROSes-CNN (23), which extracts raw sequence encoding features; ROSes-NN (24), which predicts protein function based on sequence information; and ROSes-XGBoost (25), which uses ensemble machine learning for functional classification. In total, 7,689 genes belong to 26 different ROSes types, constituting 2.18% of potential ROSes.

### Evolutionary analysis module

#### Analysis of Ka, Ks, and Ka/Ks ratios

We performed multiple sequence alignments using MUSCLE software v3.8.155. Subsequently, we generated maximum likelihood trees with 1,000 bootstrap replicates using IQ-TREE (26) version 2.1.4-beta. The Ka and Ks were calculated using ParaAT (27), and the ratio of non-synonymous to synonymous substitutions (Ka/Ks ratio) was computed using the KaKs Calculator (28). Density plots of Ka and Ks values were generated using R software.

#### Simulation of evolutionary analysis

To further investigate ROSes, we conducted significance mapping (mutation mapping) (29) analysis on ROSes. The construction of the significance map is as follows: at each position in the protein sequence, we substituted each amino acid with different amino acids, generating mutant sequences. These mutant sequences were then fed into a 0th-level model, which outputted the predicted probability of the mutant sequences being ROSes. We performed this process for each amino acid substitution at every position. Therefore, the dimensions of the significance map were *L* × 20, where *L* represents the length of the sequence. At each position in the protein sequence, we substituted each amino acid with different amino acids, generating mutant sequences, and then fed these mutant sequences into the model, which predicted the probability of the mutant sequences being ROSes.

#### Network analysis

The topology and robustness of gene networks were analyzed by conducting alignments among all sequences. This step enabled the extraction of similarity scores between each pair of genes directly from the outcomes of blast alignments. Leveraging these extracted similarity scores, we proceeded to construct a similarity matrix wherein each row and column represented a gene, and the values within the matrix denoted the similarity scores between corresponding pairs of genes. Utilizing the igraph (30) library, we then transformed this similarity matrix into a network, enabling us to meticulously compute and analyze the network’s topological features. This network was visualized using the Gephi (31).

The robustness of the network can be viewed as the ability of the network to maintain the same performance when nodes in the network are influenced by random factors and intentional disruptions. The survivability of the network can be measured by changes in the topological structural features of the network. We selected the relative size of the largest connected subgraph (RS) and the relative efficiency after node failure (RE) as metrics to measure the survivability of the network (32).


$$RS=\frac{N^{t}}{N}$$

Here, *N* represents the number of nodes in the largest connected subgraph, and *N* represents the total number of nodes in the network. As nodes in the network are attacked, the network splits into several subgraphs, and the relative size of the largest connected subgraph gradually decreases until nodes are no longer interconnected, eventually becoming isolated nodes. In this study, various attack strategies were developed to further understand the failure processes of the network in different scenarios, including random failures and targeted attacks. These strategies can be summarized as follows: (i) The random node attack strategy (33) involves the indiscriminate selection of nodes for removal from the network. The randomness of this strategy is essential for simulating the effects of unpredictable environmental factors or random gene disruptions. (ii) The maximum degree priority node attack strategy (34) focuses on nodes with the highest degree scores for targeted attacks. This strategy is predicated on the hypothesis that nodes with the highest connectivity are critical for maintaining network cohesion. (iii) The maximum PageRank priority node attack strategy (35) attacks based on nodes with the highest PageRank score. The PageRank algorithm assesses the relative importance of nodes within the network, with higher scores indicating nodes that are not only highly connected but also linked to other highly connected nodes. This strategy tests the network’s resilience against the loss of its most influential components.

## SUPPLEMENTAL MATERIAL

Fig. S1 msphere.00346-24-s0001.tiff Random combinations of ROSes.

Supplemental Tables msphere.00346-24-s0002.xlsx Tables S1 to S5.

ASM does not own the copyrights to Supplemental Material that may be linked to, or accessed through, an article. The authors have granted ASM a non-exclusive, world-wide license to publish the Supplemental Material files. Please contact the corresponding author directly for reuse.
