# Supplementary figures and images for: Hierarchical multi-task deep learning-assisted construction of human gut microbiota reactive oxygen species-scavenging enzymes database
Source: mSphere. 2024 Jul 12;9(7):e00346-24. doi: 10.1128/msphere.00346-24 (PMC11288040; doi:10.1128/msphere.00346-24)

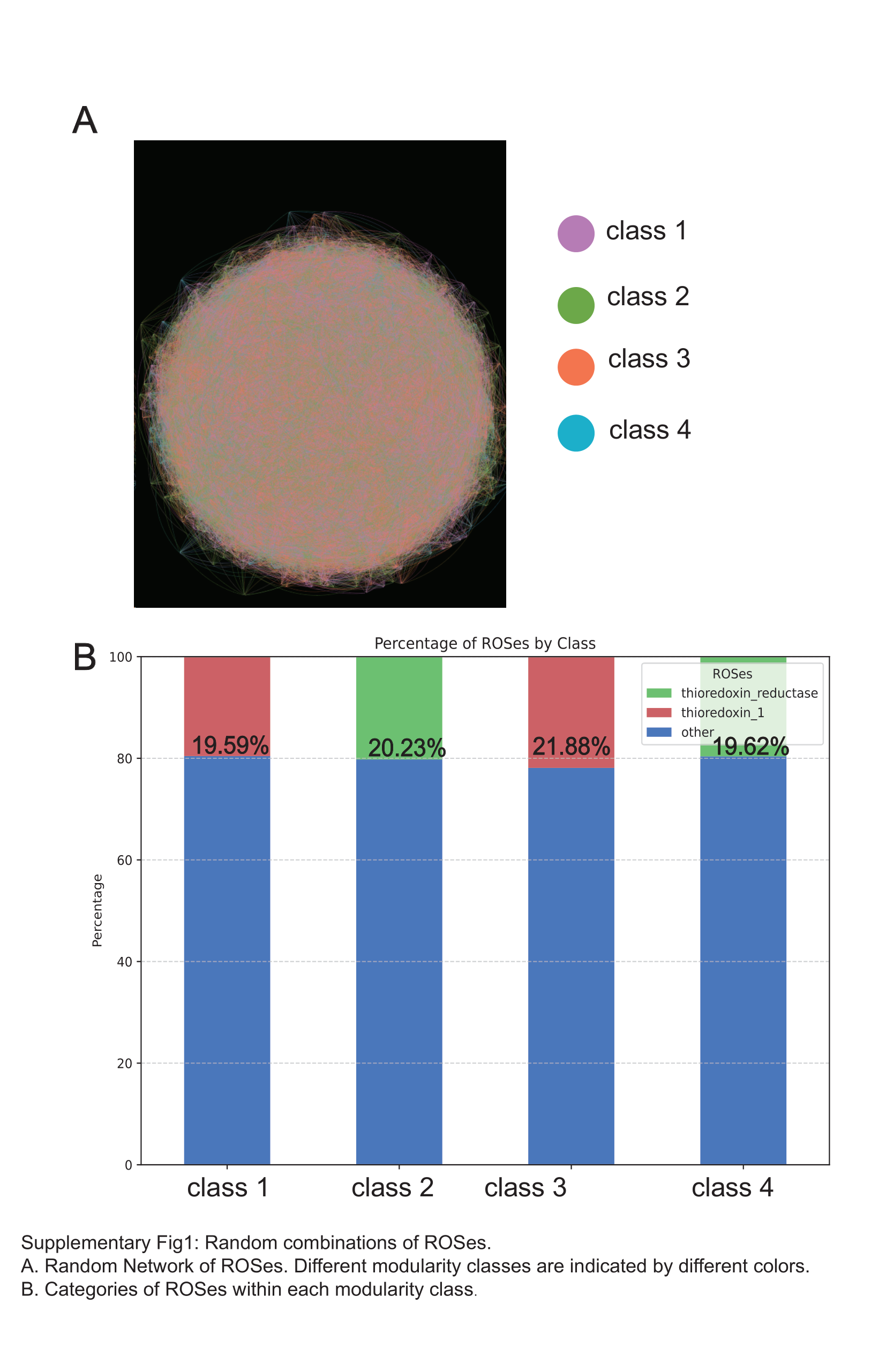

Supplement: Fig. S1 — Random combinations of ROSes. [file msphere.00346-24-s0001.tiff]
